# Yield of early postoperative computed tomography after frontal ventriculoperitoneal shunt placement

**DOI:** 10.1371/journal.pone.0198752

**Published:** 2018-06-19

**Authors:** Maria Kamenova, Jonathan Rychen, Raphael Guzman, Luigi Mariani, Jehuda Soleman

**Affiliations:** Department of Neurosurgery, University Hospital of Basel, Basel, Switzerland; Mayo Clinic Minnesota, UNITED STATES

## Abstract

Despite being widely used, ventriculoperitoneal (VP) shunt placement is a procedure often associated with complications and revision surgeries. Many neurosurgical centers routinely perform early postoperative cranial computer tomography (CT) to detect postoperative complications (e.g., catheter malposition, postoperative bleed, over-drainage). Because guidelines are lacking, our study aimed to evaluate the yield of early routine postoperative CT after shunt placement for adult hydrocephalus. We retrospectively reviewed 173 patients who underwent frontal VP shunting for various neurosurgical conditions. Radiological outcomes were proximal catheter malposition, and ventricular width in preoperative and postoperative imaging. Clinical outcomes included postoperative neurological outcome, revision surgery because of catheter malposition or other causes, mortality, and finally surgical, non-surgical, and overall morbidity. In only 3 (1.7%) patients did the early routine postoperative CT lead to revision surgery. Diagnostic ratios for CT finding 1 asymptomatic patient who eventually underwent revision surgery per total number to scan were 1:58 for shunt malposition, 1:86 for hygroma, and 1:173 for a cranial bleed. Five (2.9%) patients with clinically asymptomatic shunt malposition or hygroma underwent intervention based on early postoperative CT (diagnostic ratio 1:25). Shunt malposition occurred in no patient with normal pressure hydrocephalus and 2 (40%) patients with stroke. Lower preoperative Evans’ Index was a statistically significant predictor for high-grade shunt malposition. We found a rather low yield for early routine postoperative cranial CT after frontal VP-shunt placement. Therefore, careful selection of patients who might benefit, considering the underlying disease and preoperative radiological findings, could reduce unnecessary costs and exposure to radiation.

## Introduction

Ventriculoperitoneal (VP) shunt placement is a standard procedure for the treatment of hydrocephalus for various conditions like normal pressure hydrocephalus (NPH), intracranial hemorrhage, aneurysmal subarachnoid hemorrhage (aSAH), meningitis, tumor, or trauma [1,2,3,4,5,6]. Despite being widely used, VP shunting is still plagued with complications that develop in 20 to 40% of the patients [7,8,9,10]. These often require revision surgeries that can increase morbidity and detrimentally affect quality of life [10,11,12].

Several complications are especially important risks after VP placement. First, malposition of the proximal catheter can sometimes necessitate the patient undergo a revision surgery [9]. Several adjunctive intraoperative tools, such as intraoperative ultrasound [13] or neuronavigation [14,15,16], have been proposed to help improve position accuracy. Second, over or under drainage can often be conservatively managed by implanting an adjustable pressure valve [9]. Lastly, bleeding complications (e.g., intraventricular or intracerebral hemorrhage) seem to be rare and seldom clinically significant [17].

Many neurosurgical centers routinely perform cranial computed tomography (CT) in the early postoperative period after VP shunt placement to detect postoperative complications such as catheter malposition, postoperative bleed, or shunt over-drainage causing hygromas or chronic subdural hematomas. Yet, guidelines are lacking and questions remains about whether imaging is clinically relevant or if neurological findings alone are reliable for detecting early complications. In this retrospective study, we evaluated the yield of early postoperative cranial CT after VP shunting for adult hydrocephalus.

## Methods

We retrospectively reviewed 173 consecutive patients who underwent frontal VP shunt placement between February 2013 and June 2016 at our institution. The study protocol was approved by the local ethics committee (EKNZ, Basel, Switzerland). Diagnoses of symptomatic hydrocephalus of various underlying diseases were confirmed by cranial CT or magnetic resonance imaging (MRI). The VP shunt was placed through a frontal approach into the lateral horn aiming toward the foramen of Monro in all patients, except one who received a temporal shunt for the treatment of a trapped temporal horn. In all patients a programmable valve was implanted.

Radiological outcome defined position of the ventricular catheter seen on the first postoperative CT imaging based on the grading system developed by Yim et al. The four grades defined catheter tip position as follows: *Grade 1*: terminates optimally in the ipsilateral frontal horn or third ventricle; *Grade 2*: terminates in the contralateral frontal horn; *Grade 3*: terminates in a non-target cerebrospinal fluid (CSF) space; and *Grade 4*: terminates in brain parenchyma.^16^ For our analysis, shunt malposition was defined as grade 3 and 4.

Ventricular width on early and late radiological follow-up imaging was compared with the preoperative findings using the Evans’ Index (EI) [18] and by measuring the temporal pole unilaterally. EI represents a ratio of the transverse diameter of the anterior horns to the internal diameter of the skull [18] and is mostly used for defining ventricular enlargement in NPH patients. Timing of CT scanning included three periods: preoperative 36 ± 62 days (range 0–472 days) before surgery, early follow up (i.e., during hospitalization) 3 ± 1.8 days after surgery, and late follow up 137 ± 261 days after surgery. All 173 patients received an early postoperative CT scan, while in 148 of them (85.5%) a late one was also performed. Clinical outcome measures included postoperative clinical outcome defined as improved, unchanged, or worse; revision surgery because of catheter malposition or other causes (e.g., intracranial bleed); mortality; and finally surgical or non-surgical and overall morbidity.

### Statistical analysis

Correlation between shunt malposition grade and clinical outcome, revision surgery for catheter malposition, surgical and overall morbidity, and mortality was analyzed using a contingency table and calculating the Fisher’s exact or Chi-square test. In patients who underwent revision surgery for malposition, postoperative bleeding, or over-drainage leading to hygroma or chronic subdural hematoma, we analyzed whether these complications would have been (or were) detected without CT scanning. We then estimated the ratio of patients who would benefit from a scan for each group separately and for all combined. Mean EI values and temporal pole measurements were compared in cases with and without shunt malposition by univariate and multivariate analysis. If any parameter remained significant after multivariate analysis, a receiver operating characteristic (ROC) analysis determined a discrimination point and its sensitivity and specificity (based on the optimal clinical applicability) for diagnosis of shunt malposition. For all other parameters, contingency tests were performed using Fisher’s exact test; other calculations performed included independent or paired samples t-test. All statistical analyses were computed using SPSS Statistics Version 21.0 (IBM Corp, 2012). A p value of <0.05 was considered significant.

## Results

### Radiological outcome

Among 173 patients who had VP shunts, postoperative CT scanning detected shunt malposition grades 2, 3, or 4 in 36 (20.8%) patients, of whom 6 (16.7%) patients then underwent revision surgery. Specifically, revision surgeries were performed in 1 of 27 (3.7%) patients with Grade 2 malposition, 1 (3.3%) of 3 patients with Grade 3 malposition, and 4 (66.7%) of 6 patients with Grade 4 malposition (Table 1). In 3 cases of shunt malposition no revision surgery was done, due to initial bad clinical condition, a palliative situation in two cases and improvement of neurology in one case (Table 1). In only 3 (1.7%) of our 173 patients did early routine postoperative CT findings indicate revision surgery. In 4 (2.3%) patients with postoperative hygroma caused by overshunting (Table 2), 2 (1.2%) had no apparent clinical symptoms and CT findings identified over drainage as cause. Of 4 (2.3%) patients who developed bleeding, 2 were associated with VP shunt insertion (i.e., chronic subdural hemorrhage in a traumatic brain injury patient after simultaneous cranioplasty, 1 discrete intraparenchymal bleed with no neurological or surgical consequences) (Table 3), and 2 occurred 1 month after surgery or were not associated with the VP shunt insertion.

**Table 1:** Clinical summary of VP shunt malposition in grade 2 with revision surgery and all grade 3 or 4 patients.

No.	Underlying diagnosis	Malposition grade	Clinical symptoms	Revision surgery	How malposition detected
1	Traumatic brain injury	2	GCS declined postop from 14 to 7	yes	Clinical symptoms
2	SAH	3	No new symptoms	yes	cCT
3	Stroke	4	Confused, agitated, headache	yes	Clinical symtoms
4	Stroke	4	No new symptoms	yes	cCT
5	Meningitis	4	No new symptoms	yes	cCT
6	SAH	4	Fluctuations of vigilance	yes	Clinical symptoms
7*	SAH	3	No new symptoms	no	cCT
8	Cavernoma bleed	3	No new symptoms	yes”	cCT
9*	TBI	4	No new symptoms	no	cCT
10°	Obstructing tumor	4	Neurological testing improved	no	cCT

cCT = cranial computer tomography; GCS: Glasgow coma scale; SAH: subarachnoid hemorrhage; postop: postoperatively

* no shunt revision surgery because of initial poor clinical condition (GCS <10) and palliative situation.

” shunt was removed due to infection

° no shunt revision after postoperative improvement in clinical exam

**Table 2:** Data of patients with hygromas due to VP shunt overdrainage.

No.	Underlying diagnosis	Clinical symptoms	Treatment	Treatment due to clinical symptoms or cCT
1	IVH	Initially no symptoms. Early postoperative CT showed small hygromas. 2 months later, confusion with progression of the hygromas prompted treatment.	Ligation of the shunt. After 2 weeks, ligation removed and valve pressure increased	clinical symptoms
2	Decompression of malignant infarction	Light headaches	Raised valve pressure	clinical symptoms
3	TBI	vegetative state	Raised valve pressure	cCT
4	IVH	No clinical symptoms	Raised valve pressure	cCT

cCT = cranial computer tomography; IVH: intraventricular hemorrhage; TBI: traumatic brain injury; postop: postoperatively

**Table 3:** Data of patients with postoperative bleeding.

Nr	Underlying diagnosis	Type of bleeding	Clinical symptoms	Treatment	Treatment due to clinical symptoms or cCT
1	TBI	Chronic SDH	No symptoms and CT on postop day 1 was unremarkable. On postoperative day 6, decline of GCS prompted a second cCT, which showed bleeding.	Burr hole drainage	clinical symptoms
2	Obstructing meningioma	Acute SDH	Found in her house in coma, 1 month after VP shunt surgery	none	clinical symptoms
3	NPH	Discrete IPH	None	none	cCT
4	Cavernoma	Re-bleed of cavernoma	Bradycardia, reduced vigilance, headache	Decompression and hematoma evacuation	clinical symptoms

Nr: number; VP: ventriculoperitoneal; cCT: cranial computer tomography; IVH: intraventricular hemorrhage; TBI: traumatic brain injury; NPH: normal pressure hydrocephalus; SDH: subdural hematoma; IPH: intraparenchymal hemorrhage; postop: postoperatively

Early routine postoperative CT detected 1 (0.6%) asymptomatic postoperative bleed, which had no therapeutic consequences.

Diagnostic ratios, that is, CT finding 1 asymptomatic patient who eventually underwent revision surgery per total number to scan, were estimated as 1:58 for shunt malposition, 1:86 for hygroma, and 1:173 for a cranial bleed. (Table 4). None of the patients underwent shunt revision based on the results of the late CT.

**Table 4:** Absolute risk reduction (ARR) and diagnostic ratio for early postoperative cranial CT of various radiological factors.

	Radiological Factor	N	ARR % (95% CI)	Ratio (95% CI)
**Malposition**	Clinically silent malposition and ReOR	3	1.7 (-0.2–3.7)	1:58 (27.2–473.7)
	Reoperation due to malposition	6	3.5 (0.7–6.2)	1:29 (16.1–134.8)
	Clinically silent malposition grade 3 or 4	7	4.0 (1.1–7.0)	1:25 (14.3–90.1)
	All grade 3 or 4 malposition	9	5.2 (1.9–8.5)	1:20 (11.7–52.8)
**Hygroma**	Clinically silent hygroma due to	2	1.2 (-0.4–2.7)	1:86 (36.0–226.3)
	overdrainage			
	Hygroma due to overdrainage	4	2.3 (0.07–4.5)	1:44 (22.0–1376.9)
Bleeding	Clinically silent bleed associated with	1	0.6 (-0.5–1.7)	1:173 (58.6–181.3)
	VP Shunt insertion			
	Postop bleed associated with VP shunt	2	1.2 (-0.4–2.7)	1:86 (36.0–226.3)
	insertion			

N: number of patients; CI: confidence interval; postop: postoperatively; VP: ventriculoperitoneal

Significant differences were noted for preoperative (0.36 ± 0.08), early postoperative (0.35 ± 0.08), and late postoperative EI (0.33 ± 0.07) (p<0.001, paired sample t-test). No significant differences were noted for these times related to temporal horn measurements (21.4 ± 75.8, 14.9 ± 48.2, and 10.6 ± 7.3, respectively). Patients with shunt malposition (grades 3 and 4) showed significantly lower preoperative (0.28 ± 0.08) and late postoperative (0.25 ± 0.05) EI values than patients without malposition (preoperative 0.36 ± 0.08, late postoperative 0.33 ± 0.07; p<0.001 and p = 0.048, respectively). After multivariate analysis showed only preoperative EI remained significantly different, ROC analysis of area under the curve (AUC) was 0.86 yielding a good test result, showing statistical significance (p<0.001, standard error 0.04). A discrimination point of a preoperative EI 0.285 was chosen, showing 85% sensitivity and 67% specificity for shunt malposition.

### Clinical outcome

Patient sex, side of VP shunt placement, preoperative American Society of Anesthesia (ASA) grade had no influence on shunt malposition (Table 5). All 3 patients who underwent placement under neuronavigation had a postoperative shunt position grade of 1; however, underlying disease was significantly associated with shunt malposition (Table 5). Specifically, shunt malposition occurred less often in normal pressure hydrocephalus (p 0.01), but was more common in stroke (p<0.001) and obstructive tumor (p 0.03) (Table 5).

**Table 5:** Comparison of cohort demographics between patients with and without shunt malposition.

Variable	No malposition (n, %) n = 164	Malposition (n, %) n = 9	p value
Sex (female) n (%)	72 (43.9)	4 (44.4)	0.62
Side (right) n (%)	132 (80.5)	5 (55.6)	0.09
Navigation (yes) n (%)	3 (1.8)	0 (0)	0.85
ASA grade			0.36
1	3 (1.9)	0 (0)	
2	30 (18.6)	0 (0%)	
3	118 (73.3)	9 (7.1)	
4	10 (100)	0 (0)	
Underlying disease			*0*.*01*
NPH	68 (41.5)	0 (0)	*0*.*01*
TBI	20 (12.2)	1 (11.1)	
SAH	33 (20.1)	3 (33.3)	
Tumor	8 (4.9)	2 (22.2)	*0*.*03*
Meningitis	5 (3.0)	1 (11.1)	
IVH	11 (6.7)	0 (0)	
PTC	2 (1.2)	0 (0)	
Malignant stroke	3 (1.8)	2 (22.2)	*<0*.*001*
AS	1 (0.6)	0 (0)	
Syrinx	1 (0.6)	0 (0)	
Postoperatively	11 (6.7)	0 (0)	
Idiopatic	1(0.6)	0 (0)	

ASA: american society of anesthesiology; NPR: normal pressure hydrocephalus; TBI: traumatic brain injury; SAH: subarachnoid hemorrhage; IVH: intraventricular hemorrhage; PTC: pseudotumor cerebri; AS: aqueductal stenosis

## Discussion

In our 173 consecutive patients with hydrocephalus caused by various diseases, we quantified a 1.7% yield for early postoperative imaging after VP shunting, specifically 3 patients underwent revision surgery for shunt malposition based solely on early routine postoperative CT. The ratio for finding 1 asymptomatic patient with proximal shunt malposition who eventually underwent revision surgery was 1:58 overall; 1:86 and 1:173 for hygroma due to over drainage (1.2%) or bleeding (0.6%), respectively; and 1:35 together for shunt malposition, hygroma, or bleeding. Lower preoperative EI was a factor statistically predictive for shunt malposition. A cutoff point of 0.285 showed a sensitivity of 85% and specificity of 67% for shunt malposition. Patients with shunt malposition had a significantly worse clinical outcome, and significantly higher rates of surgical morbidity, overall morbidity, and reoperation rates.

### Justification for early routine postoperative CT

Several considerations are important for identifying which patients should undergo routine scanning after any surgical procedure. First and foremost, the diagnostic and therapeutic benefits of the scans should outweigh any potential harm. Second, the scans should regularly detect pathologies or complications that might occur but are not easily detected clinically or do not cause obvious clinical symptoms. Lastly, the timing of scanning should maximize the amount of information gained.

Our findings address the paucity of published information regarding the ideal timing for and yield of cranial CT (early or late) after VP shunt placement. Although the rational for CT scanning is classically to assess ventricular size and rule out shunt malposition, hygroma, or SDH caused by over drainage or postoperative bleeding, we found a low 2.9% probability for detecting a clinical complication that would need intervention. Rates (diagnostic ratio) included clinically silent malposition in 1.7% of overall cases (1:58), clinically silent over drainage in 1.2% of cases (1:86), and with 1 clinically silent bleeding with no need for surgical intervention, estimated a 0.6% rate for detection by early routine CT (1:173).

The ratio 1:35 (1 detected silent complication needing intervention: 35 placed VP shunts) underlines the question, “Is early routine postoperative CT justified?’ Additionally, we do not know how many of these patients would have eventually developed clinical symptoms that would then require treatment. However, if not detected and treated early, these complications could lead to longer treatment courses with higher costs, or increased or more severe permanent morbidity or even mortality. As expected, early postoperative CT did not play a role in assessing postoperative ventricular size because it was too early to expect a significant change.

In our cohort, the largest group of patients (41.5%) who underwent VP shunting suffered from NPH; none showed shunt malposition or over drainage on the early postoperative CT, and 1 NPH patient with a small asymptomatic intraparenchymal hemorrhage was treated conservatively. Therefore, routine early postoperative imaging in this group might be redundant if clinical symptoms are lacking. In contrast, shunt malposition was more common in patients suffering from obstructive tumors (20%, 50% with grade 4, 50% with grade 2), malignant stroke (40%, all grade 4), or meningitis (16.5%, all grade 4). In these cases, malposition was more common because of the distorted cerebral anatomy caused by stroke or tumor. In the case of meningitis, brain edema and small or even slit ventricles can impede catheter insertion into the ventricle in the often young patients affected. Therefore, the use of early postoperative CT is reasonable and even recommended in these patients.

Along with the underlying diagnosis, preoperative radiological findings may play a crucial role in selecting patients who might benefit from early CT scans. In our cohort, a preoperative EI <0.285 was a significant predictor for catheter malposition. Thus, in these patients, early postoperative cranial CT might be justified.

Neuronavigation, which can help avoid catheter malposition, is not used routinely at our institution. None of our 3 patients treated with neuronavigation showed shunt malposition. Although robust evidence for the usage of neuronavigation does not exist, some retrospective studies showed good results with very low rates of shunt malposition [15,16,19]. Nonetheless, navigation might be associated with longer operation times and additional invasiveness through cranial fixation [20,21,22]. On the other hand, electromagnetic navigation does not need pinning and is therefore less invasive. The use of cranial navigation seems reasonable in patients with a higher risk of shunt malposition (e.g., stroke, meningitis, tumor, low EI) but perhaps not as valuable in patient with low risk (i.e., NPH). As neuronavigation becomes routine, the yield for early routine postoperative CT will likely further decline.

### Study limitations

This retrospective study was subject to all the limitations of data collection inherent in such studies. The course of treatment (surgical versus observation) for silent shunt malposition was decided by the treating surgeon and might have led to a selection bias. In addition, some of the patients did or did not undergo shunt revision due to the imaging findings alone, which might limit our results. Although EI [23] does not seem to be the ideal method for estimating ventricular width in patients with NPH, volumetric measurements, which may be more precise, were not performed [23]. Nonetheless, the preoperative EI proved to be a good predictor for patients prone to shunt malposition. Additionally, the findings related to shunt malposition in our study can only apply to patients treated with frontally placed shunts. Further studies are needed for patients with occipitally placed shunts and those who undergo shunt procedures with the help of neuronavigation. Finally, an early postoperative CT scan might be useful as a baseline image for comparison with later follow-up imaging.

## Conclusions

Our findings of low yields for routine early postoperative CT after frontal VP shunt placement address the paucity of information in the literature regarding the ideal timing for and yield of early imaging after shunting. Careful selection of the patients who might benefit based on the underlying disease and preoperative radiological findings might reduce unnecessary costs and exposure to radiation. With no NPH patient having an early CT scan that showed shunt malposition or overdrainage, imaging in this group might be redundant if clinical symptoms are lacking. In contrast, early postoperative CT might be reasonable or even recommended in patients with stroke, tumor, or meningitis when malposition is more common because of distorted cerebral anatomy.

## Supporting information

S1 Table Patient data file. (SAV)
